# Unravelling Kikuchi-Fujimoto Disease: A Unique Presentation of Recurrent Aseptic Meningitis in a District Hospital Setting

**DOI:** 10.7759/cureus.74897

**Published:** 2024-12-01

**Authors:** Baraa Chkir, Aroon Sivaji

**Affiliations:** 1 Urology, Royal Albert Edward Infirmary; Wrightington, Wigan and Leigh NHS Foundation Trust, Wigan, GBR; 2 Emergency Medicine, Whiston Hospital; Mersey and West Lancashire Teaching Hospitals NHS Trust, Whiston, GBR

**Keywords:** aseptic meningitis, benign histiocytic lymphadenitis, case report, kikuchi-fujimoto disease, lymphadenopathy, rare

## Abstract

Kikuchi-Fujimoto disease (KFD) is a rare, self-limiting, and ultimately benign condition characterised by localised lymphadenopathy. The association of KFD with aseptic meningitis is even more uncommon. We report a case of KFD accompanied by aseptic meningitis in a 31-year-old male who initially presented with lethargy, night sweats, axillary lymphadenopathy, and oral ulcers. Initial differential diagnoses included Lyme disease and lymphoma, which were subsequently ruled out. The patient presented again 10 days later with a severe headache. Neurological examination revealed negative Kernig and Brudzinski signs and normal motor power, tone, and reflexes. Lumbar puncture (LP) indicated normal glucose and white cell count. A lymph node biopsy confirmed the diagnosis of Kikuchi’s disease with aseptic meningitis. This case underscores the importance of considering KFD in patients presenting with lymphoma-like symptoms.

## Introduction

Kikuchi-Fujimoto Disease (KFD), also known as histiocytic necrotising lymphadenitis, was independently described by Kikuchi and Fujimoto in 1972 in Japan [1,2]. The identification of KFD was significant because it delineated a unique form of lymphadenitis that mimics serious conditions like lymphoma and systemic lupus erythematosus (SLE), potentially leading to misdiagnosis and inappropriate treatments [3]. Over the past five decades, an understanding of KFD has evolved, but it remains a diagnostic challenge due to its rare occurrence and non-specific clinical presentation.

Histopathologically, KFD is characterised by necrotising lymphadenitis with abundant karyorrhectic debris and proliferation of histiocytes (immune cells responsible for phagocytosis and antigen presentation) [4]. The necrosis occurs without granulocytic infiltration, meaning neutrophils are absent, which helps differentiate KFD from bacterial infections and other lymphadenitides [3].

Epidemiologically, KFD predominantly affects young adults between 20 and 35 years of age, with a female-to-male ratio of approximately 4:1 [5,6]. While initially reported in Asia, particularly Japan, China, and South Korea, cases have been documented worldwide, including in North America, Europe, and the Middle East [7]. The higher prevalence in Asian populations may be attributed to genetic predispositions, environmental factors, or heightened clinical awareness in these regions.

Clinically, KFD typically presents with tender cervical lymphadenopathy (the most common feature, occurring in 50-98% of cases [8]), fever and night sweats, constitutional symptoms, such as fatigue, weight loss, and malaise, skin manifestations like rashes which occurs in about 10-40% of cases [9], and arthralgia and myalgia.

The disease usually follows a benign, self-limiting course over one to four months, but recurrences occur in up to 3-4% of patients [10].

Misdiagnosis is a significant concern with KFD due to its resemblance to lymphoma and SLE [3,11]. Patients may undergo unnecessary invasive procedures or receive inappropriate treatments like chemotherapy or immunosuppressive therapy, which carry substantial risks and increase healthcare costs. Misdiagnosis can also lead to psychological distress for patients [11].

Neurological complications are rare in KFD, with aseptic meningitis being the most commonly reported neurological manifestation, occurring in approximately 2-7% of cases [12]. Patients may present with severe headache, neck stiffness, photophobia, and other meningeal signs, but cerebrospinal fluid (CSF) analysis typically reveals no bacterial pathogens. While the pathogenesis is not fully understood, it may involve immune-mediated mechanisms or viral triggers [13].

Understanding the pathogenesis of KFD is crucial. Two main hypotheses exist:

Infectious agents: Viruses like Epstein-Barr virus (EBV), human herpesvirus 6, and parvovirus B19 have been implicated, but no definitive causal relationship has been established [14].

Autoimmune response: The disease's histological similarities to SLE and the occasional progression of KFD patients to develop SLE suggest an autoimmune mechanism [15].

We report this case to highlight the diagnostic challenges of KFD presenting with aseptic meningitis in a non-Asian male patient and to emphasise the importance of considering KFD in the differential diagnosis to prevent misdiagnosis and inappropriate management.

## Case presentation

A 31-year-old, Caucasian, male tree surgeon presented to the emergency department with a six-week history of lethargy impacting his daily activities, drenching night sweats occurring almost nightly, painful left axillary lymphadenopathy, and multiple oral ulcers interfering with eating and speaking. He also reported a pustular rash on his forearms and arthralgia affecting his elbows and hands, characterised by intermittent pain without swelling or redness. There was no history of recent travel, tick bites, exposure to tuberculosis, or family history of autoimmune diseases.

Physical examination revealed the following:

Vital signs: Temperature of 38.5 °C, blood pressure of 134/86 mmHg, heart rate of 90 beats per minute, respiratory rate of 16 breaths per minute, and oxygen saturation of 98% on room air

On general examination, the patient appeared fatigued but alert and oriented. There was no evidence of pallor or jaundice. Axillary examination revealed left axillary lymphadenopathy, which was tender, firm, and mobile. Oral examination revealed ulcers on the buccal mucosa and tongue. There was a pustular rash on forearms, non-pruritic with no discharge on skin examination. Musculoskeletal examination showed tenderness over elbows and hands without swelling or redness. There was a full range of motion. 

Laboratory findings showed leucopenia with neutropenia and lymphopenia, elevated lactate dehydrogenase (LDH) and ferritin levels, and mildly elevated erythrocyte sedimentation rate (ESR) and C-reactive protein (CRP) (Table 1).

**Table 1:** Initial laboratory tests

Lab Tests	Results	Normal Values
Haemoglobin (Hb)	148 g/L	130–180 g/L
Mean Corpuscular Volume (MCV)	95 fL	80–100 fL
White Blood Count (WBC)	3.1 x 10^9^/L	4.0–11.0 x 10^9^/L
Neutrophils	1.3 x 10^9^/L	1.8–7.5 x 10^9^/L
Lymphocytes	0.5 x 10^9^/L	1.0–4.0 x 10^9^/L
C-Reactive Protein (CRP)	6.6 mg/L:	<5 mg/L:
Erythrocyte Sedimentation Rate (ESR)	12 mm/h	1–7 mm/h
Lactate Dehydrogenase (LDH)	600 U/L	135–225 U/L
Sodium	139 mmol/L	133–146 mmol/L
Potassium	4.1 mmol/L	3.5–5.3 mmol/L
Urea	4.9 mmol/L	2.5–7.8 mmol/L
Creatinine	73 μmol/L	59–104 μmol/L

Initial differential diagnosis and management

Given his occupation and symptoms, Lyme disease was considered. Lymphadenopathy and elevated LDH raised suspicion for lymphoma. Intravenous ceftriaxone (2 g once daily) was initiated empirically for suspected Lyme disease pending serology, which came back negative. After symptomatic improvement, he was discharged.

Second presentation

Ten days later, he returned with a severe generalised headache rated 10/10, throbbing in nature, worsened by movement, not relieved by over-the-counter analgesics and right upper quadrant abdominal pain associated with nausea and vomiting. He had no photophobia or neck stiffness.

His vitals were: temperature: 37.8°C, blood pressure: 125/78 mmHg, heart rate: 88 beats per minute, respiratory rate: 18 breaths per minute, and oxygen saturation: 99% on room air.

The neurological examination was normal with a Glasgow Coma Scale (GCS) of 15/15. The cranial nerves examination was intact. He had normal tone and power (5/5 in all muscle groups) with symmetrical reflexes. There were no sensory deficits with normal cerebellar function. He also had negative Kerning and Brudzinski signs indicating no signs of meningeal. The abdominal examination revealed mild right upper quadrant tenderness with no hepatosplenomegaly.

Investigations included a meningitis panel, which was negative for common pathogens (Neisseria meningitidis, Listeria monocytogenes, Streptococcus pneumoniae, cytomegalovirus, herpes simplex virus, Varicella-Zoster virus, human herpesvirus, and Cryptococcus). Lumbar puncture revealed an opening pressure of 18 cm H₂O (Normal: 10-20 cm H₂O) with a clear appearance, white cell count of 5 cells/μL (Normal: 0-5 cells/μL) with predominantly lymphocytes, no red blood cells, protein of 1.46 g/L (Normal: 0.15-0.45 g/L), and normal glucose of 3.8 mmol/L. No organisms were detected in the gram stain and culture.

Further tests, including blood cultures, antistreptolysin O titres, HIV, Epstein-Barr virus (EBV), hepatitis B and C, cytomegalovirus (CMV), parvovirus, Lyme serology, antinuclear antibodies (ANA), extractable nuclear antigens (ENA), complement levels (C3 and C4), and angiotensin-converting enzyme (ACE) levels, were all negative or within normal limits.

Imaging studies, including ultrasound of the axillary region and computed tomography of the thorax, abdomen, and pelvis, revealed generalised small-volume lymphadenopathy (<1 cm) with no masses or organomegaly.

He was then discharged with a plan to review him in an outpatient clinic.

Third presentation

Six days after discharge, he presented with recurrent low-grade fevers (38 °C) but no headaches. Given the persistent lymphadenopathy and systemic symptoms, a core biopsy of the left axillary lymph node was performed, which revealed the following:

Macroscopy

Two pieces of core tissue measuring 20 mm and 22 mm and two tiny fragments measuring 1 mm each.

Microscopy

The core biopsies revealed a lymph node. The lymph node showed scattered lymphoid follicles with germinal centres. The inter-follicular areas were expanded by variable-sized lymphoid cells with sinuses showing histiocytes. There were areas showing necrosis with abundant nuclear debris. The areas of necrosis were followed by histiocytes, including foamy macrophages. Well-formed granulomata were, however, not seen. The peri-lymphoid connective tissue also showed fat necrosis and groups of histiocytes along with fibrosis.* *

There were areas showing course granular dark brown pigment (anthracotic/tattoo pigment). Necrotising lymphadenitis was seen. 

The differential diagnosis included Kikuchi's lymphadenitis, lupus lymphadenitis, infections (including tuberculosis, fungal, parasitic) and less likely lymphoproliferative process. Cytokeratin AE1/AE3 and Melan-A were negative. CD20 was focally positive in scattered cells likely neoplastic cells. CD3 was non-contributory and S-100 was positive in dendritic cells. CD68 was diffusely positive in histiocytes. 

ZN, modified ZN, Grocot, and DPAS stains were negative for acid-fast bacilli and fungal organisms. This is typical of necrotising lymphadenitis, likely Kikuchi's lymphadenitis, provided lupus lymphadenitis is ruled out by an appropriate clinical workup. The conclusion drawn by a left axillary lymph node biopsy was necrotising lymphadenitis.

The patient declined a bone marrow biopsy. The patient was managed with supportive care, including analgesia and antipyretics. Corticosteroids were considered but not initiated due to clinical improvement. At the two-week follow-up, he remained asymptomatic with no recurrence of symptoms. Blood counts and liver enzymes normalised.

## Discussion

Kikuchi-Fujimoto disease is a rare cause of lymphadenitis, particularly in non-Asian populations. Its aetiology remains unclear, but both infectious and autoimmune mechanisms have been proposed [14,15]. The disease's rarity and non-specific presentation often lead to misdiagnosis.

Clinical presentation and diagnostic challenges

Our patient's symptoms of lymphadenopathy, fever, night sweats, rash, and arthralgia were consistent with KFD but also overlapped with lymphoma and autoimmune diseases like SLE [3,11]. The presence of oral ulcers and arthralgia raised suspicion for SLE, but serological tests were negative.

Neurological manifestations in KFD are uncommon. Aseptic meningitis occurs in approximately 2-7% of cases based on case series and reviews [12]. The pathogenesis may involve immune-mediated vasculitis or an autoimmune response triggered by infectious agents [13].

Importance of early diagnosis

Early and accurate diagnosis of KFD is crucial to prevent unnecessary treatments. Lymph node biopsy remains the gold standard for diagnosis [4]. Histopathology shows necrotising lymphadenitis with abundant karyorrhectic debris, proliferation of histiocytes, plasmacytoid dendritic cells, and absence of neutrophils and eosinophils [4]. These features help distinguish KFD from lymphoma and SLE.

Management

The management of KFD is primarily supportive, as the disease is usually self-limiting [16]. Non-steroidal anti-inflammatory drugs (NSAIDs) can be used for symptomatic relief. Corticosteroids are reserved for severe or persistent cases, especially with extranodal manifestations like aseptic meningitis [17]. Our patient improved without corticosteroids, indicating that conservative management can be sufficient in some cases.

Follow-up and prognosis

The recurrence of KFD is rare but documented in up to 4% of cases [10]. Long-term follow-up is recommended due to the potential development of SLE or other autoimmune conditions [15]. Our patient remained asymptomatic at follow-ups, and no autoimmune disease developed.

Significance of reporting this case

Reporting this case adds to the limited literature on KFD presenting with aseptic meningitis, especially in non-Asian males. It highlights the importance of considering KFD in the differential diagnosis of young patients with lymphadenopathy and neurological symptoms, regardless of ethnic background. Increased awareness can lead to timely diagnosis, appropriate management, and avoidance of unnecessary interventions.

## Conclusions

Kikuchi-Fujimoto disease should be considered in young patients presenting with lymphadenopathy and systemic symptoms, especially when initial investigations for infections and malignancies are negative. Although rare, aseptic meningitis can be a manifestation of KFD. Early lymph node biopsy is vital for accurate diagnosis. Management is generally supportive, but corticosteroids may be considered in severe or recurrent cases. Awareness of this condition can prevent misdiagnosis and inappropriate treatment.
